# ZFP281 recruits polycomb repressive complex 2 to restrict extraembryonic endoderm potential in safeguarding embryonic stem cell pluripotency

**DOI:** 10.1007/s13238-020-00775-x

**Published:** 2020-08-18

**Authors:** Xin Huang, Nazym Bashkenova, Jihong Yang, Dan Li, Jianlong Wang

**Affiliations:** 1 Department of Medicine, Columbia Center for Human Development, Columbia University Irving Medical Center, New York, NY 10032 USA; 2 Department of Cell, Developmental and Regenerative Biology, Black Family Stem Cell Institute, Icahn School of Medicine at Mount Sinai, New York, NY 10029 USA

**Dear Editor,**

Cell-fate decisions are governed by comprehensive gene-regulatory programs. During the preimplantation development, at least two waves of cell fate decisions are made while the cells gradually lose their totipotency (Schrode et al., 2013). The first decision involves the spatial separation of outer-residing trophectoderm (TE) cells from inner cell mass (ICM) in E3.5 mouse blastocyst. The second decision involves gene expression refinements and active cell sorting within the ICM that ultimately results in epiblast (EPI) cells, residing deep within the ICM, and the primitive endoderm (PrE) cells comprising a monolayer of blastocoel-facing cells at the surface of the ICM (Schrode et al., 2013). OCT4, SOX2, and NANOG are master transcription factors (TFs) essential for the formation and maintenance of the pluripotent ICM cells and their *in vitro* counterparts mouse embryonic stem cells (ESCs). On the other hand, GATA4, GATA6, and SOX17 are master TFs of the PrE cells and their *in vitro* counterparts extraembryonic endoderm stem cells (XENs). Only naïve ESCs are capable of deriving both primed epiblast stem cells (EpiSCs) and XEN cells *in vitro* (Cho et al., 2012). Primed EpiSCs cannot derive XEN-like cells, suggesting that ESCs and EpiSCs have different levels of developmental potential (Cho et al., 2012). Understanding the mechanism of pluripotent state transition *in vitro* provides insights into dynamic control of *in vivo* developmental transition of embryonic potency while simultaneously preparing for the transition to the somatic lineages.

We have investigated the functions of zinc finger protein 281 (ZFP281) in ESC maintenance and ESC-to-EpiSC differentiation (Fidalgo et al., 2011; Fidalgo et al., 2016; Huang et al., 2017). Interestingly, ZFP281 was also reported to be expressed in XEN cells (Wang et al., 2008). However, roles of ZFP281 in the maintenance of XENs and during ESC-to-XEN differentiation are elusive. To address this, we firstly examined the expression of ZFP281 and the pluripotency and PrE factors in ESCs, EpiSCs, and XENs. ZFP281 protein is highly abundant at similar levels in pluripotent ESCs and EpiSCs, which is in contrast with a much lower level of ZFP281 protein in XENs (Fig. S1A). However, the mRNA levels of *Zfp281* are comparable in all tested cell lines (Fig. S1B), indicating that ZFP281 is regulated at both transcriptional and post-transcriptional levels. Using WT and *Zfp281*^−/−^ ESCs (Fidalgo et al., 2011), we investigated the effects of ZFP281 in ESC-to-XEN differentiation following a well-established protocol (Cho et al., 2012) to convert ESCs into stable XEN-like cells (referred to hereafter as chemical-induced XEN cells, or cXENs, Fig. 1A). Briefly, feeder-free ESCs were treated with retinoic acid (RA) and activin for 2 days, then replated on MEF feeders to further support the culture of cXENs. While RA treatment induced significant differentiation of ESCs at Passage 1 (P1), stellate and refractile XEN-like colonies emerged from both WT and *Zfp281*^−/−^ ESCs after replating on MEF feeders (Fig. 1B, P2, white arrows). However, for the cells derived from WT ESCs, after a few days of culture on MEF, compact and dome-shape (ESC-like, red arrows in Fig. 1B) colonies reemerged and became dominant at P2 when the cells were confluent. This is probably because that treatment of RA for 2 days, while pushing ESCs to exit pluripotency, is not enough to commit to a XEN fate. In addition, MEF feeders may provide additional factors such as LIF to reestablish pluripotency. In contrast, dome-shaped colonies were rarely seen in *Zfp281*^−/−^ ESC-derived cXENs at P2, indicating a more committed XEN fate. When cells were further cultured for one more passage (P3) on feeder-free plates, almost all *Zfp281*^−/−^ ESC-derived cXENs showed stellate and highly refractile XEN morphology (Fig. 1B, P3, white arrows), without the need of picking XEN colonies, a necessary step in regular cXEN derivation protocol (Cho et al., 2012). Next, we collected RNAs at P0, P1, P3 (Fig. 1A), when cells were cultured on feed-free plates to avoid RNA contamination from MEF cells, for qRT-PCR analysis. First, we observed that *Zfp281* mRNA was activated by RA and activin treatment at P1 in WT cells (Fig. 1C). We also found steady downregulation of pluripotency genes (*Oct4*, *Nanog*, *Sox2*) and upregulation of PrE genes (*Gata4*, *Gata6*, *Sox17*) in *Zfp281*^−/−^ ESC-derived cXENs (Fig. 1D). In contrast, WT ESCs experienced an initial downregulation of pluripotency genes and upregulation of endoderm genes at P1, followed by expression reversal of these same genes back to initial (P0) levels at P3 (Fig. 1D), likely due to the reappearance of ESC-like colonies in culture (Fig. 1B, WT cells, P3). Immunostaining of pluripotency factor NANOG and PrE factor GATA6 was performed. RA and activin treatment activated GATA6 expression in both WT and *Zfp281*^−/−^ ESCs, but GATA6 expression was much higher in *Zfp281*^−/−^ ESCs than that in WT ESCs (Fig. 1E, P1, merged panel). At P3, most WT ESC-derived cells were NANOG positive, while most *Zfp281*^−/−^ ESC-derived cells were GATA6 positive (Fig. 1E). In addition, we rescued *Zfp281*^−/−^ ESCs with exogenous expression of ZFP281 (Fig. S2A). Compared to the mRNA expression of pluripotency (*Oct4*, *Nanog*, *Sox2*) and PrE (*Gata4*, *Gata6*, *Sox17*) genes from *Zfp281*^−/−^ ESCs, the *Zfp281*-rescue line phenocopied that of the WT ESCs in cXEN differentiation (Fig. S2B). To gain a global view of gene expression changes of WT and *Zfp281*^−/−^ ESCs in ESC-to-XEN differentiation, we investigated the transcriptome changes by performing RNA-sequencing (RNA-seq) analysis of the collected RNAs. There were 1,857 and 528 significantly (fold-change > 2, T-test *P*-value < 0.01) up- and down-regulated genes, respectively, in XENs relative to ESCs (Fig. 1F; Table S1). Expression of the ESC-signature genes (*n* = 528) was significantly lower in *Zfp281*^−/−^ ESC-derived cXENs at P3 (Fig. 1G), and expression of the XEN-signature genes (*n* = 1,857) was significantly higher in *Zfp281*^−/−^ ESC-derived cXENs at P1 and P3 (Fig. 1H) compared to WT cells. In addition, the same trend was observed by comparing the expression ESC- and XEN-signature genes between *Zfp281*^−/−^ and *Zfp281*-rescue ESCs in cXEN differentiation (Fig. S2C). These results are consistent with and expand on the expression of those selected pluripotency and PrE master genes tested by qRT-PCR (Figs. 1D and S2B). The *Zfp281*^−/−^ ESC-derived cXENs could maintain XEN morphology and expression of PrE genes for a long period of time (30 days) without the need of picking up XEN colonies (Figs. 1A, S2D, and S2E). Taken together, our data indicate that ZFP281 is a barrier in ESC-to-XEN differentiation and that *Zfp281*KO greatly facilitates XEN fate commitment.

**Figure 1:**
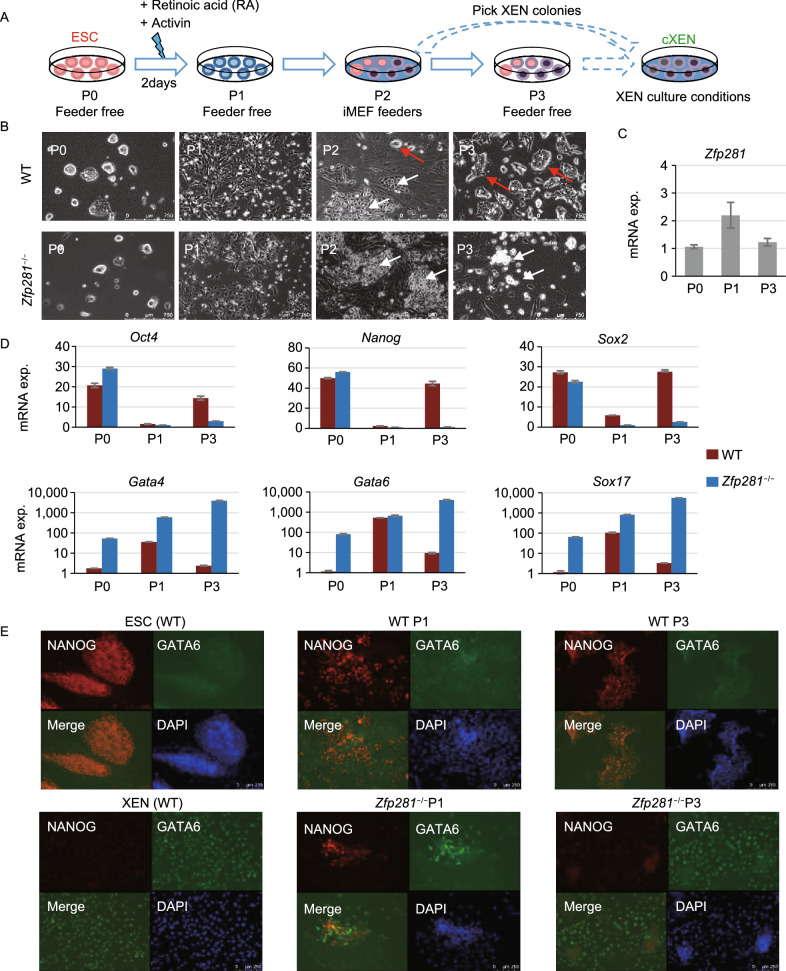

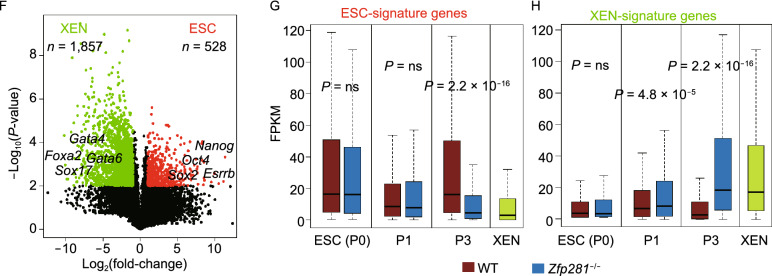
**ZFP281 functions as a barrier in ESC-to-XEN differentiation**. (A) A schematic plot of ESC-to-XEN differentiation *in vitro*. To avoid contamination of irradiated MEF feeders, RNAs were collected at P0 (passages 0), P1 and P3 (feeder-free) for qRT-PCR analysis. (B) Phase contrast microscope images of WT and *Zfp281*^−/−^ ESC-derived cXEN cells at P0-P3. White and red arrows indicate the XEN-like and ESC-like colonies, respectively. (C and D) qRT-PCR analysis of *Zfp281* (C), pluripotency (*Oct4*, *Nanog*, *Sox2*) and PrE (*Gata4*, *Gata6*, *Sox17*) (D) transcripts in WT and *Zfp281*^−/−^ ESC-derived cXENs at P0, P1, and P3. (E) Immunostaining of NANOG and GATA6 at P1 and P3 in ESC-to-XEN differentiation. WT ESCs and XENs were used as positive controls for NANOG and GATA6 staining, respectively. (F) A volcano plot of differentially expressed genes (DEGs, fold-change > 2, T-test *P*-value < 0.01) between ESCs and XENs. DEGs highly expressed in ESC and XEN were defined as ESC-signature genes and XEN-signature genes, respectively. (G and H) Box plots for the expression of ESC-signature genes (G) and XEN-signature genes (H) in WT and *Zfp281*^−/−^ ESCs in cXEN differentiation. *P*-value was from a Mann-Whitney test

Since PrE master regulators GATA4 and GATA6 are also expressed in the embryonic definitive endoderm (DE), we investigated the function of ZFP281 in ESC-to-DE formation, following a published protocol (Fig. S3A) (Gouon-Evans et al., 2006). Briefly, ESCs were subjected to 2 days of embryonic body (EB) differentiation, followed by resuspension and activin treatment for another 2 or 3 days. We confirmed that a DE-specific marker, *Foxa2*, was markedly elevated in WT DE cells (Fig. S3B). Strikingly, while *Zfp281*^−/−^ ESCs were capable of forming EBs at day 2 (Fig. S3C), as previously reported (Fidalgo et al., 2011), these cells gradually died out during EB-to-DE transition (Fig. S3C and S3D). Since DE is a descendant lineage of EPI, our data suggest that failed DE differentiation of *Zfp281*^−/−^ ESCs may be due to the requirement of ZFP281 in EPI development, which is supported by our earlier study that ZFP281 is indispensable for the ESC-to-EpiSC transition (Fidalgo et al., 2016).

Inquiring into the molecular mechanism by which ZFP281 represses the PrE program in ESC-to-XEN differentiation, we first found a striking (>50 fold) upregulation of PrE gene transcripts *Gata4*, *Gata6*, *Sox17* in *Zfp281*^−/−^ ESCs relative to WT ESCs (Fig. 1D), suggesting that ZFP281 may transcriptionally repress the PrE targets. We have previously found that ZFP281 interacts and recruits polycomb repressive complex 2 (PRC2) to repress the bivalent lineage genes in ESCs (Huang et al., 2017). By processing public RNA-seq datasets (Hon et al., 2014; Cruz-Molina et al., 2017), we found that KO of PRC2 catalytic subunit *Eed* significantly increased expression of the XEN-signature genes and the PrE regulators *Gata4*, *Gata6*, *Sox17* (Fig. S4A and S4B). TET1 is another known partner of ZFP281 (Fidalgo et al., 2016) with dual functions in transcriptional regulation (Wu et al., 2011); however, it does not function as a transcriptional repressor of PrE genes in ESCs (Fig. S4C and S4D). In line with a prior study demonstrating that the embryonic tissues are relatively more hypo-methylated than extraembryonic tissues in pre-implantation embryos (Senner et al., 2012), we observed that CpG islands around promoters of PrE genes (*Gata4*, *Gata6*, *Sox17*) were hypo-methylated in ESCs compared to XENs, suggesting that the repression of PrE genes by ZFP281 is not through DNA methylation (Fig. S4E). Therefore, we performed ChIP-sequencing (ChIP-seq) analysis of ZFP281 and the PRC2 component SUZ12 in ESCs and XENs. In ESCs, ZFP281 and SUZ12 co-bind at promoters of PrE genes *Gata4* and *Gata6*, but not at promoters of pluripotency genes *Oct4* or *Nanog* (Fig. 2A). In XENs, ZFP281 binds to promoters of *Gata4* and *Gata6*, but not *Oct4* or *Nanog* (Fig. 2A). Moreover, SUZ12 binds to promoters of neither pluripotency genes nor PrE genes, likely due to the fact that ZFP281 doesn’t bind to pluripotency genes in XENs (Fig. 2A), and that PrE genes *Gata4* and *Gata6* are highly expressed in XENs, respectively. The ChIP-seq results were verified by ChIP-qPCR analyses of the selected pluripotency and PrE genes in ESCs and XENs (Fig. 2B and 2C). In addition, SUZ12 ChIP signals decrease in *Zfp281*^−/−^ ESCs compared to that in WT cells (Fig. 2D), suggesting that ZFP281, at least partially, recruits PRC2 to the promoters of PrE genes in ESCs.

**Figure 2:**
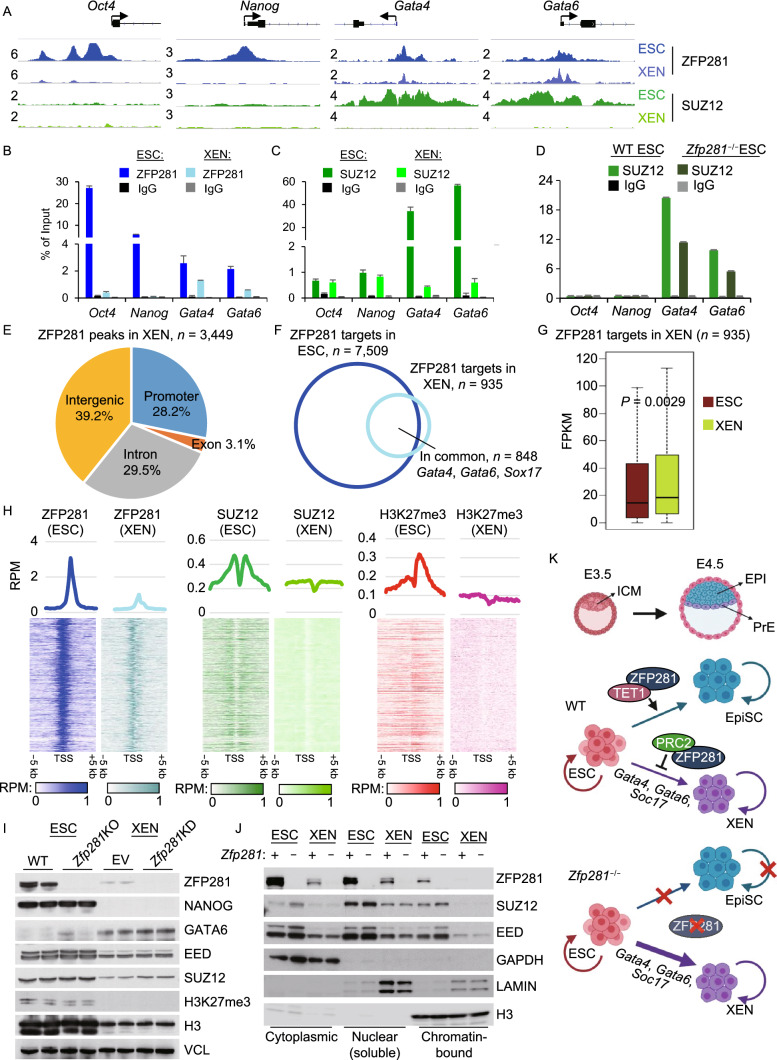
**ZFP281 recruits PRC2 for transcriptional repression of PrE master regulators in ESC-to-XEN differentiation**. (A) ChIP-seq tracks of ZFP281 and SUZ12 chromatin-binding at *Oct4*, *Nanog*, *Gata4*, and *Gata6* promoters in ESCs and XENs. Track heights of different ChIP-seq data were normalized to the same mapped reads per million total reads (RPM). (B and C) ChIP-qPCR for the ZFP281 (B) and SUZ12 (C) chromatin-binding at *Oct4*, *Nanog*, *Gata4*, and *Gata6* promoters. (D) ChIP-qPCR for the SUZ12 chromatin-binding in WT and *Zfp281*^−/−^ ESCs at *Oct4*, *Nanog*, *Gata4*, and *Gata6* promoters. (E) Distribution of ZFP281 ChIP-seq peaks in XENs. Promoter was defined as a peak distance to TSS less than 1 kbp. (F) Overlap of the ZFP281 targets (peak distance to TSS < 1 k bp) in ESCs and XENs. (G) Relative expression of the ZFP281 targets in XENs (*n* = 935) to that in ESCs. *P*-value is from a Mann-Whitney test. (H) Mean intensity plots (RPM) and heatmaps of ZFP281, SUZ12, and H3K27me3 ChIP-seq data in ESCs and XENs enriched at TSSs of the ZFP281 target genes in XENs (*n* = 935). H3K27me3 ChIP-seq in ESCs were curated from (Cruz-Molina et al., 2017). (I) Expression of ZFP281, SUZ12, EED, and H3K27me3 in ESCs and XENs. Two KO clones (2.6 Null, 3.34 Null) of *Zfp281*^−/−^ ESCs and KD by two shRNAs (sh#1, sh#3) were used to deplete *Zfp281* in ESCs and XENs, respectively. VCL (Vinculin) served as the protein loading control. (J) Expression of ZFP281, SUZ12, and EED in different subcellular fractions in ESCs and XENs. *Zfp281* was depleted by KO in ESCs and by KD in XENs. GAPDH, LAMIN, and H3 (Histone3) served as the control proteins in cytoplasmic, nuclear (soluble) and chromatin-bound fractions, respectively. (K) Depiction of the working model. During the *in vivo* ICM to EPI/PrE differentiation and *in vitro* ESC to EpiSC/XEN differentiation, ZFP281 functions as a barrier in ESC-to-XEN (ICM-to-PrE) differentiation by recruiting PRC2 for transcriptional repression of PrE genes *Gata4*, *Gata6*, and *Sox17*. ZFP281 is dispensable for self-renewal of ESCs and XENs, but is indispensable for ESC-to-EpiSC differentiation through a ZFP281-TET1 partnership and for self-renewal of EpiSCs (Fidalgo et al., 2016)

To understand the potential roles of ZFP281 in XENs, we processed the genome-wide ChIP profiles of ZFP281 in XENs. A total of 3,449 ZFP281 peaks were identified, 28.2% (972/3,449) of which were located at promoters (within 1 kb of TSS) (Fig. 2E). Compared to the number of ZFP281 peaks in ESCs, fewer ZFP281 peaks were identified in XENs, majority of which (80.6%, 2,779/3,449) were shared with those in ESCs (Fig. S5A). Consistently, fewer ZFP281 target genes in XENs were identified, majority of which (90.5%, 846/935) were also identified as ZFP281 targets in ESCs, including the PrE genes *Gata4*, *Gata6*, *Sox17* (Fig. 2A and 2F). Expression of the ZFP281 target genes (*n* = 935) in XENs were significantly higher than that in ESCs (Fig. 2G), likely due to their repression by PRC2, which is recruited by ZFP281, in ESCs but not in XENs (Fig. 2H). We also observed a general low ChIP signals of SUZ12 and H3K27me3 in XENs (Figs. 2H and S5B), which is consistent with a previous finding that H3K27me3 is scarce in XENs (Rugg-Gunn et al., 2010). To investigate the effects of ZFP281 in XEN self-renewal, we performed short-hairpin RNAs (shRNAs) mediated knockdown (KD). While an efficient KD of *Zfp281* was obtained by two independent shRNAs in XENs, cell morphology and expression of PrE genes at both RNA and protein levels were not affected upon *Zfp281* KD (Fig. S6A–D), which is consistent with our findings that *Zfp281*^−/−^ cXENs can be derived from *Zfp281*^−/−^ ESCs and maintained in XEN culture (Fig. S2D). We also found that expression levels of ZFP281, PRC2 subunits EED, SUZ12, and H3K27me3 were all lower in XENs that those in ESCs (Fig. 2I). Furthermore, consistent with our ChIP-seq result of SUZ12 and H3K27me3 in XENs (Fig. 2H), the chromatin-bound fractions of ZFP281, SUZ12, and EED were barely observed in XENs (Fig. 2J). Of note, depletion of ZFP281 doesn’t affect expression of PRC2 and H3K27me3 (Fig. 2I) or the overall intensity of chromatin-bound PRC2 (Fig. 2J) in ESCs or XENs. Together, these data suggest that ZFP281/PRC2 repress the promoters of PrE genes in ESCs and during ESC-to-XEN transition, and that ZFP281 is dispensable for XEN self-renewal.

In summary, this study uncovers a novel role of ZFP281 in recruiting PRC2 for transcriptional repression of the PrE program encompassing the master regulators GATA4, GATA6, and SOX17 in restricting ESC-to-XEN differentiation. As a result, *Zfp281*^−/−^ ESCs exhibit a lineage tendency towards the PrE program with an enhanced potential of XEN differentiation upon chemical treatment (Fig. 2K). ZFP281 is also dispensable for self-renewal of XENs. These findings are in marked contrast with the requirements of the ZFP281-TET1 partnership for ESC-to-EpiSC differentiation (Fig. 2K) and for EpiSC self-renewal (Fidalgo et al., 2016). Maintenance of pluripotent cells *in vitro* depends on sustained activation of extracellular signaling that controls specific gene expression programs, such as LIF/STAT3 signaling for survival and self-renewal of naive ESCs and FGF signaling for maintenance of primed EpiSCs. Nodal signaling activator activin is supplied with FGF ligand in EpiSC culture, which however cannot sustain the maintenance of *Zfp281*^−/−^ EpiSCs (Fidalgo et al., 2016). *Zfp281*^−/−^ ESCs do form EBs, but these EBs undergo cell death when medium was supplied with activin in EB-to-DE differentiation (Fig. S3). Therefore, ZFP281 is likely involved in the downstream events of activin/FGF signaling to maintain cell survival in early development (Huang et al., 2017). FGF signaling is also essential to form PrE lineage and for *in vitro* ESC-to-XEN differentiation (Cho et al., 2012), suggesting other targets than ZFP281 may be downstream of FGF signaling in promoting PrE differentiation from ICM cells.

Recently, XEN-like cells were revealed as an intermediate stage in an alternative route of somatic reprogramming by Yamanaka factors (Parenti et al., 2016) or small molecules (i.e., chemical induced pluripotent stem cells, CiPSCs) (Zhao et al., 2015). Interestingly, transitional colonies that co-expressed XEN master genes and pluripotency-associated genes must be captured, if CiPSCs were induced from XEN-like cells (Zhao et al., 2015). This intermediate stage in CiPSC reprogramming may be similar to the cXENs emerged after a short period (48 h) and low concentration (0.01 µmol/L) of RA treatment in ESC-to-XEN differentiation (Fig. 1E, co-expression of GATA6 and NANOG at P1). It is known that RA can activate expression of PrE master genes through RAR/RXR signaling (Chatagnon et al., 2015). Importantly, these intermediate cXENs are plastic and can be reverted back to a pluripotent state without the need of additional chemicals (in XEN medium, with serum, no LIF, and on MEF feeders). Moreover, *Zfp281*^−/−^ decreases the potential of the intermediate cXENs (P1) to reestablish the pluripotency state (Fig. 1), suggesting a potentially necessary role of ZFP281 in CiPSC induction, which awaits experimental confirmation. Finally, our study reveals that XENs are also characteristic of additional features such as low PRC2 activity and lack of the repressive histone mark H3K27me3 (Fig. 2H–I), making them a unique population of multipotent stem cells to study cell reprogramming, plasticity, and fate transition.

## Electronic supplementary material

Below is the link to the electronic supplementary material.

Supplementary material 1 (PDF 888 kb)

Supplementary material 2 (XLSX 544 kb)
