# Correction to: ZFP281 recruits polycomb repressive complex 2 to restrict extraembryonic endoderm potential in safeguarding embryonic stem cell pluripotency

**DOI:** 10.1007/s13238-020-00817-4

**Published:** 2021-01-16

**Authors:** Xin Huang, Nazym Bashkenova, Jihong Yang, Dan Li, Jianlong Wang

**Affiliations:** 1 Department of Medicine, Columbia Center for Human Development, Columbia University Irving Medical Center, New York, NY 10032 USA; 2 Department of Cell, Developmental and Regenerative Biology, Black Family Stem Cell Institute, Icahn School of Medicine at Mount Sinai, New York, NY 10029 USA

## Correction to: Protein Cell https://doi.org/10.1007/s13238-020-00775-x

In the original publication the labelling in middle and bottom panels of Fig. 2K is published incorrectly as “Soc17”. The correct labeling is available in this correction as “Sox17”.

**Figure:**
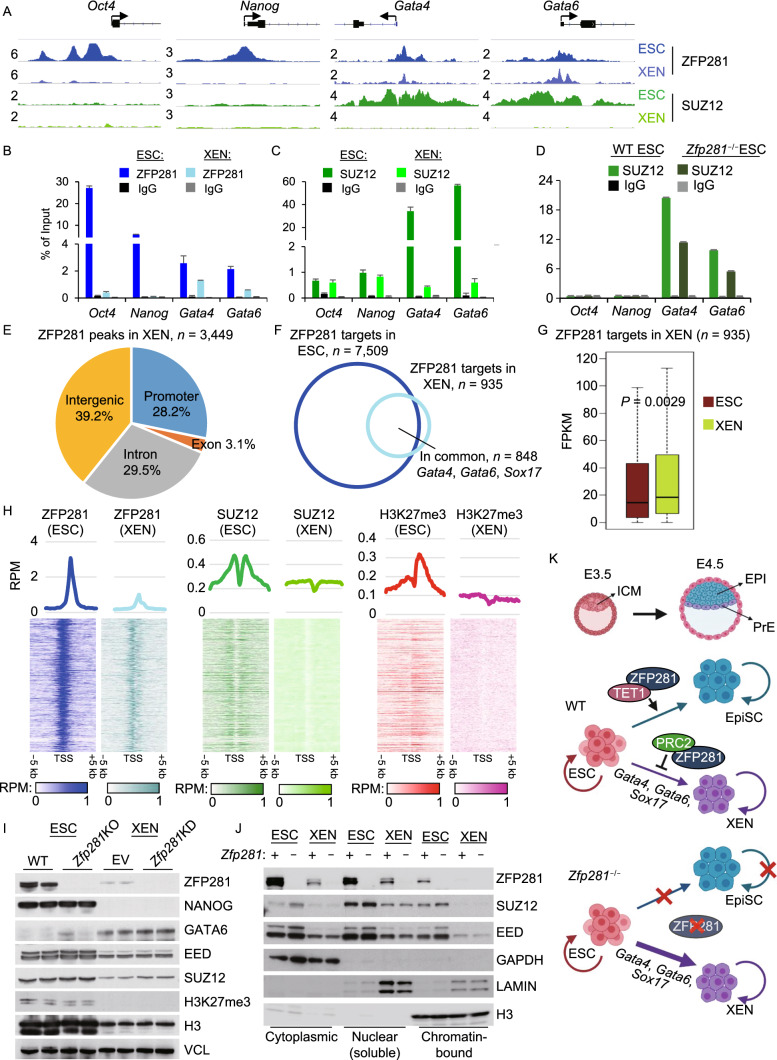
**ZFP281 recruits PRC2 for transcriptional repression of PrE master regulators in ESC-to-XEN differentiation**. (A) ChIP-seq tracks of ZFP281 and SUZ12 chromatin-binding at *Oct4*, *Nanog*, *Gata4*, and *Gata6* promoters in ESCs and XENs. Track heights of different ChIP-seq data were normalized to the same mapped reads per million total reads (RPM). (B and C) ChIP-qPCR for the ZFP281 (B) and SUZ12 (C) chromatin-binding at *Oct4*, *Nanog*, *Gata4*, and *Gata6* promoters. (D) ChIP-qPCR for the SUZ12 chromatin-binding in WT and *Zfp281*^−/−^ ESCs at *Oct4*, *Nanog*, *Gata4*, and *Gata6* promoters. (E) Distribution of ZFP281 ChIP-seq peaks in XENs. Promoter was defined as a peak distance to TSS less than 1k bp. (F) Overlap of the ZFP281 targets (peak distance to TSS < 1 k bp) in ESCs and XENs. (G) Relative expression of the ZFP281 targets in XENs (*n* = 935) to that in ESCs. *P*-value is from a Mann-Whitney test. (H) Mean intensity plots (RPM) and heatmaps of ZFP281, SUZ12, and H3K27me3 ChIP-seq data in ESCs and XENs enriched at TSSs of the ZFP281 target genes in XENs (*n* = 935). H3K27me3 ChIP-seq in ESCs were curated from (Cruz-Molina et al., 2017). (I) Expression of ZFP281, SUZ12, EED, and H3K27me3 in ESCs and XENs. Two KO clones (2.6 Null, 3.34 Null) of *Zfp281*^−/−^ ESCs and KD by two shRNAs (sh#1, sh#3) were used to deplete *Zfp281* in ESCs and XENs, respectively. VCL (Vinculin) served as the protein loading control. (J) Expression of ZFP281, SUZ12, and EED in different subcellular fractions in ESCs and XENs. *Zfp281* was depleted by KO in ESCs and by KD in XENs. GAPDH, LAMIN, and H3 (Histone3) served as the control proteins in cytoplasmic, nuclear (soluble) and chromatin-bound fractions, respectively. (K) Depiction of the working model. During the *in vivo* ICM to EPI/PrE differentiation and *in vitro* ESC to EpiSC/XEN differentiation, ZFP281 functions as a barrier in ESC-to-XEN (ICM-to-PrE) differentiation by recruiting PRC2 for transcriptional repression of PrE genes *Gata4*, *Gata6*, and *Sox17*. ZFP281 is dispensable for self-renewal of ESCs and XENs, but is indispensable for ESC-to-EpiSC differentiation through a ZFP281-TET1 partnership and for self-renewal of EpiSCs (Fidalgo et al., 2016)

