# Supplementary material for: ZFP281 recruits polycomb repressive complex 2 to restrict extraembryonic endoderm potential in safeguarding embryonic stem cell pluripotency
Source: Protein Cell. 2020 Aug 18;12(3):213–9. doi: 10.1007/s13238-020-00775-x (PMC7895869; doi:10.1007/s13238-020-00775-x)
Supplement: Supplementary file 1 — Supplementary material 1 (PDF 888 kb) [file 13238_2020_775_MOESM1_ESM.pdf]

METHODS

Cell Culture

WT (J1, CJ7, 3WT) and *Zfp281*$^{-/-}$ (2.6Null, 3.34Null) mouse ESCs and EpiSCs (OEC2, Episc9) were maintained as described previously (Fidalgo et al., 2016; Huang et al., 2017). XENs (XEN5, XENB1) were cultured on either irradiated MEF (iMEF) feeders with standard XEN medium (advanced RPMI 1640 supplemented with 15% FBS, 0.1 mM β-mercaptoethanol and 1% penicillin-streptomycin), or on 0.1% gelatin-coated plates with MEF-conditioned XEN medium, as described (Niakan et al., 2013).

Knockdown of Zfp281 in XENs

Two shRNAs for *Zfp281* knockdown were validated from our previous study (Fidalgo et al., 2016). Lentivirus production and infection were performed. Concentrated viral supernatants were incubated with resuspended XENs for 1 hour, then cells were diluted with fresh medium. Blasticidin (10 μg/mL) was used for selection 24 hours later. Cells were harvested after a minimal of 72 hours of viral infection.

In Vitro ESC-to-XEN Differentiation

ESCs were differentiated to XENs following a published protocol (Niakan et al., 2013). Briefly, ESCs (Passage 0) were trypsinized and cultured on gelatin-coated plates with XEN medium. Twenty-four hrs later, cells were treated with all-*trans* retinoic acid (0.01 μM) and activin A (10 ng/mL) for 48 hrs (Passage 1), then trypsinized and transferred onto iMEF feeders and XEN medium for another 3~5 days until cells were confluent (Passage 2). Cells were trypsinized again and cultured on gelatin-coated plates with XEN medium (Passage 3). XEN colonies form at passage 2 when cells were recovered on iMEF feeders. These colonies could be picked and maintained in XEN culture condition.

In Vitro ESC-to-DE Differentiation

ESCs were differentiated to definitive endoderm (DE) cells following a published protocol (Gouon-Evans et al., 2006). Briefly, ESCs (Day 0) were trypsinized and resuspended in low-attachment petri dishes with serum-free differentiation (SFD) medium to allow embryonic bodies (EBs) formation. Two days later (Day 2), EBs were

trypsinized and reaggregated in petri dishes with SFD medium supplemented with activin A (100 ng/mL) for another two (Day 4) or three (Day 5) days.

Cell Viability Assay

The Annexin V/Propidium Iodide (PI) Apoptosis Detection Kit I from BD Biosciences (BD Biosciences, NJ) was used for cell viability assay. Single-cell suspensions were analyzed on an LSRII Flow Cytometer System (BD Biosciences). Data were analyzed with FlowJo software.

Subcellular Fractionation Assay

The subcellular fractions of ESCs and XENs were extracted using the Subcellular Protein Fractionation Kit for Cultured Cells (Thermo, #78840). Briefly, about 5 x 10^6 cells were used and each subcellular fraction was collected following the standard protocol. Protein loadings were balanced according to the protein concentrations in the cytoplasmic fraction before western blot analysis.

Western Blot Analysis

Western blot analysis was performed as previously described (Huang et al., 2017). Total proteins were extracted by RIPA buffer, and balanced before running SDS-PAGE. The following primary antibodies were used: anti-ZFP281 (Santa Cruz, sc-166933), anti-OCT4 (Santa Cruz, sc-5279), anti-SUZ12 (Abcam, ab12073), anti-EED (Cell Signaling, #85322), anti-LAMIN A/C (Cell Signaling, #4777), anti-H3K27me3 (Millipore, #07-449), anti-Histone3 (Abcam, ab1791), anti-GAPDH (ProteinTech, 10494-1-AP), anti-VINCULIN (Abcam, ab129002), anti-NANOG (Bethyl, A300-397A), anti-GATA6 (R&D systems, AF1700), anti-SOX17 (R&D systems, AF1924), and anti-ACTIN (Sigma, A5441).

Immunofluorescence

Cells were grown on 0.1% gelatin-coated 24-well plates, and fixed with 4% paraformaldehyde for 15 min at room temperature (RT), permeabilized with 0.25% Triton X-100 in PBS for 5 min at RT, and blocked with 10% bovine serum albumin (BSA, Sigma, MO) for 30 min at 37 °C. Primary antibodies were incubated overnight at 4 °C in PBS with 3% BSA. The next day, samples were incubated with fluorophore-labelled appropriate secondary

antibodies for 1 hour at RT, and treated with 1 μM DAPI for nuclei staining. Images were collected on a LEICA DMI 6000 inverted microscope at 20x magnification, and image settings were maintained at constant levels for each set of images acquired per factor stained. The primary antibodies used: anti-ZFP281 (Santa Cruz, sc-166933), anti-NANOG (Bethyl, A300-397A), anti-GATA6 (R&D systems, AF1700), and anti-SOX17 (R&D systems, AF1924).

qRT-PCR

Total RNA was extracted using the RNeasy kit (Qiagen, Hilden, Germany) and cDNA was generated using qScript (Quanta, Cat# 95048). Relative expression levels were determined using the LightCycler 480 SYBR green mix (Roche, #4729749001). qRT-PCR experiments were performed on a LightCycler Real Time PCR System (Roche, Basel, Switzerland). Gene expression levels were normalized to Gapdh. Error bars indicate standard error of average expression. The primers used in this study were:

Zfp281	For:	GCACCACCGCGATGTATTACT
	Rev:	CCTTTTTGACGTTAGCGTCCTG
Oct4	For:	GTGGAGGAAGCCGACAACAATGA
	Rev:	CAAGCTGATTGGCGATGTGAG
Nanog	For:	TGGTCCCCACAGTTTGCCTAGTTC
	Rev:	CAGGTCTTCAGAGGAAGGGCGA
Sox2	For:	CTGGACTGCGAACTGGAGAAG
	Rev:	AATTTGGATGGGATTGGTGGT
Gata4	For:	CCCTACCCAGCCTACATGG
	Rev:	ACATATCGAGATTGGGGTGTCT
Gata6	For:	TTGCTCCGGTAACAGCAGTG
	Rev:	GTGGTCGCTTGTGTAGAAGGA
Sox17	For:	CGCACGGAATTCGAACAGTA
	Rev:	GTCAAATGTCGGGGTAGTTG
Gapdh	For:	ACCCAGAAGACTGTGGATGG
	Rev:	CACATTGGGGGTAGGAACAC

RNA-sequencing and Data Analysis

Total RNAs of ESCs, XENs, and intermediate cells during ESC-to-XEN differentiation were collected for RNA-seq analysis. About 0.1~1 μg total RNA from each sample was used to prepare PolyA RNA-seq libraries, and

massively parallel sequencing was performed with the HiSeq4000 platform. Paired-end 150 bp-length reads were produced.

For the RNA-seq data from this study or public resources, reads were aligned to the genome using STAR software (v2.5.3) with the default parameter settings. UCSC mouse genome sequence (mm9) and the transcript annotation were downloaded from the iGenomes site. Transcript assembly and differential expression analysis were performed using Cufflinks (v2.1.1). Assembling of novel transcripts was not allowed (-G), other parameters of Cufflinks followed the default setting. The summed FPKM (fragments per kilobase per million mapped reads) of transcripts sharing each gene_id was calculated and exported by the Cuffdiff program. A minimal FPKM of 0.1 was assigned if the expression value is less than 0.1. The differential expressed genes between ESCs and XENs were determined by $\log_2$(fold-change)>2 and T-test P-value < 0.01.

Chromatin Immunoprecipitation (ChIP), Library Preparation, Sequencing, and Data Analysis

ChIP assays were performed as described (Huang et al., 2017). Briefly, cells were cross-linked with 1% (w/v) formaldehyde for 10 min at RT, and formaldehyde was inactivated by the addition of 125 mM glycine. Sonication was performed on a Bioruptor system, with 30s ON, 30s OFF, 30 cycles, high amplitude. Chromatin extracts were immunoprecipitated by incubating with primary antibody-conjugated DynaBeads (Novex, 10003D) overnight with rotation at 4 °C. The following primary antibodies were used for ChIP: anti-ZFP281 (Abcam, ab101318), anti-SUZ12 (Active Motif, #39357), anti-H3K27me3 (Millipore, #07-449), anti-Rabbit IgG (Millipore, PP64). For ChIP-qPCR, ChIP intensity was normalized to Input. The primers used in this study were shown in Table S1.

Oct4	For:	CACTCTAGGGAAGTTCAGGGTAGG
	Rev:	TCAAAGACAGAGCCTCAGATGGA
Nanog	For:	AATGAGGTAAAGCCTCTTTTTGG
	Rev:	ACCATGGACATTGTAATGCAAA
Gata4	For:	TCACTCCCTGTAGCCCTCAC
	Rev:	GACGCCGACTCCAAACTAAG
Gata6	For:	CCAAGTTTCCCTCCCTCTTC
	Rev:	CAGCCCTAGCGGACAATAAG

ChIP libraries were prepared using the NEBNext Ultra II DNA library prep kit and index primers sets (NEB, #7645S, #E7335S) followed the standard protocol. Massively parallel sequencing was performed by Novogene Co. with the Illumina HiSeq 4000 Sequencer according to the manufacturer's protocol. Libraries were sequenced as 150 bp paired-end reads.

ChIP-seq reads were aligned to the mm9 mouse genome using the bowtie2 (v2.3.4) with parameters "-X 1000 --no-mixed --no-discordant". The aligned paired reads were exported (-F 0x04 -f 0x02) and sorted with samtools. Duplicates were removed with MarkDuplicates function in PICARD (v2.14.0) package. Bam files were converted to a binary tiled file (tdf) and visualized using IGV (v2.7.2) software. ChIP-seq peaks were determined by the MACS2 program (v.2.0.10) using the input ChIP-seq as the control data, and all other parameters followed the default settings. ChIP-seq peaks were annotated using the annotatePeaks module in HOMER program (v4.11) against the mm9 genome. A target gene of Zfp281 was defined as nearest gene's transcription start site (TSS) with a distance less than 1k bp.

DNA meDIP-seq Data Analysis

The meDIP-seq data from previously study (Senner et al., 2012) were downloaded and processed. Briefly, reads were aligned to the mm9 mouse genome using the bowtie (v1.2.3) with parameters "-m 1 -v 2 --best --strata". The aligned paired reads were exported and sorted with samtools. Bam files were converted to a binary tiled file (tdf) and visualized using IGV (v2.7.2) software. The list of CpG Island in mouse genome (mm9) was downloaded from the UCSC table browser.

SUPPLEMNTAL TABLES

Table S1. Expression of the ESC-signature and XEN-signature genes in all RNA-seq samples.

SUPPLEMNTAL FIGURE LEGENDS

Figure S1. Expression of ZFP281 in ESCs, EpiSCs, and XEN cells. (A) Protein expression levels of ZFP281, OCT4, NANOG, and GATA6 in ESC lines (J1, CJ7), EpiSC lines (OEC2, EpiSC9), and XEN line (XENB1). (B) mRNA expression levels *Zfp281*, *Oct4*, *Nanog*, and *Gata6* in the same ESCs, EpiSCs, and XEN cell lines as in (A).

Figure S1

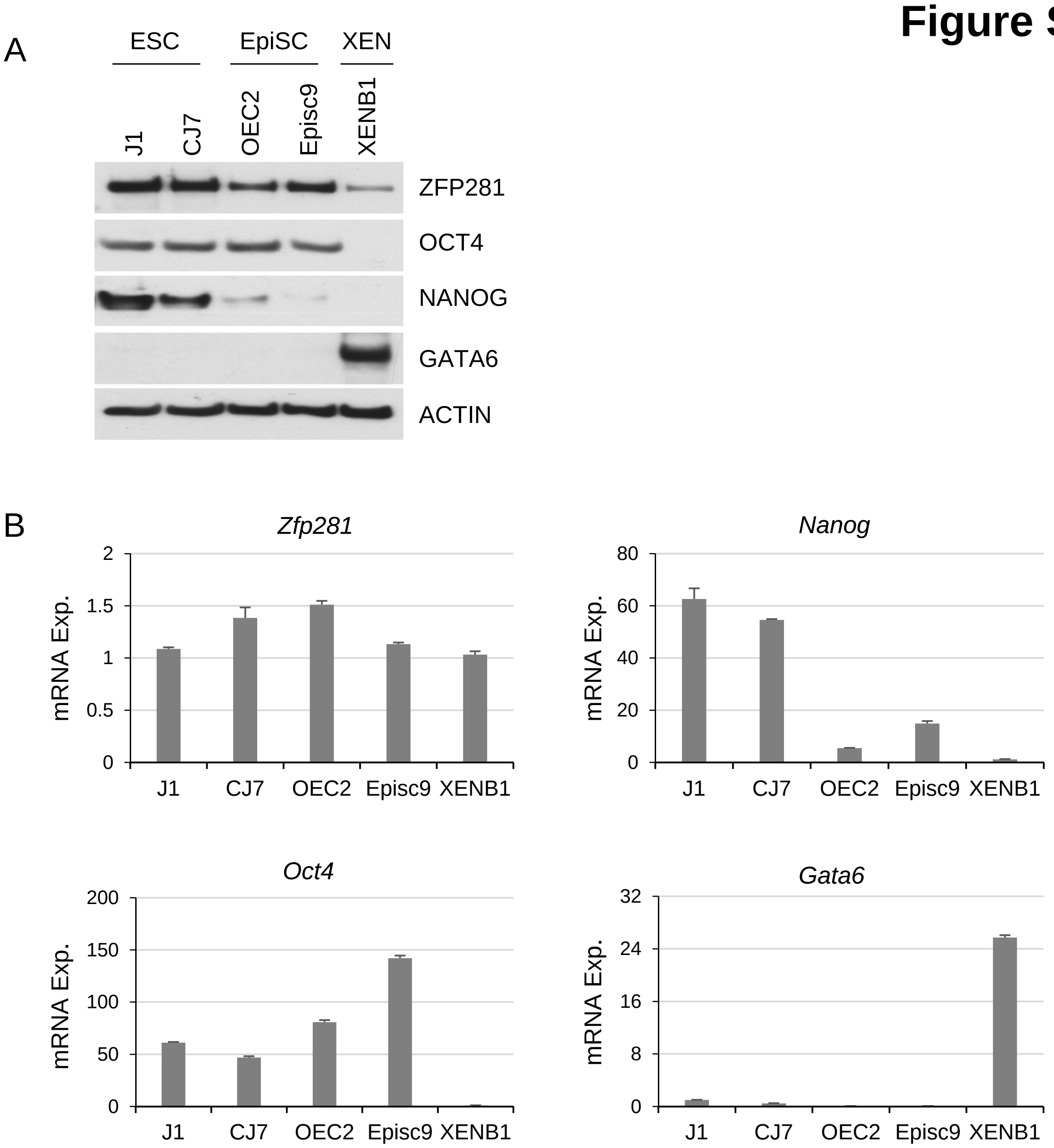
A
ESC
EpiSC
XEN
J1
CJ7
OEC2
Episc9
XENB1
ZFP281
OCT4
NANOG
GATA6
ACTIN
B
Zfp281
Nanog
Oct4
Gata6
mRNA Exp.
0
0.5
1
1.5
2
20
40
60
80
50
100
150
200
8
16
24
32
J1
CJ7
OEC2
Episc9
XENB1

Figure S2. Exogenous expression of ZFP281 rescues *Zfp281$^{-/-}$* **ESCs in XEN differentiation.** (A) Western blot analysis confirmed exogenous rescue of ZFP281 in *Zfp281$^{-/-}$* ESCs. (B) mRNA expression levels of pluripotency (*Oct4, Nanog, Sox2*) and PrE (*Gata4, Gata6, Sox17*) genes in *Zfp281$^{-/-}$* and *Zfp281*-rescued ESC-derived cXENs at P0, P1, and P3. (C) Box plots for the expression of ESC-signature genes (left) and XEN-signature genes (right) in *Zfp281$^{-/-}$* and *Zfp281*-rescue ESCs in cXEN differentiation. P-value is from a Mann-Whitney test. (D) Phase contrast microscope images of WT and *Zfp281$^{-/-}$* ESC-derived cXENs (Day 30, stable lines without clone picking). (E) mRNA expression levels of pluripotency genes (*Oct4, Nanog*) and PrE genes (*Gata4*, *Gata6*) in WT and *Zfp281$^{-/-}$* ESCs (Day 0), ESC-derived cXEN (Day 30), and XEN cell lines.

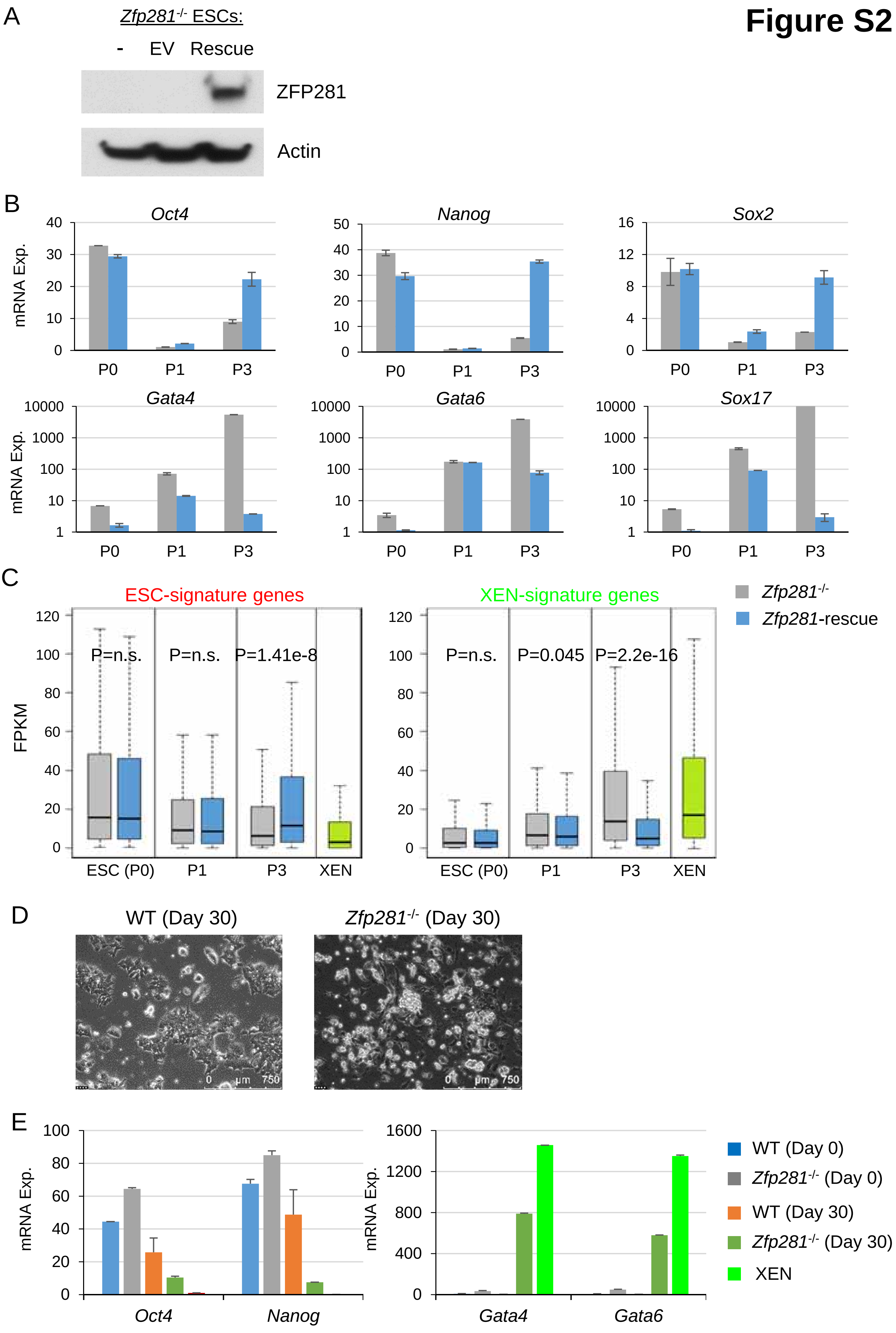

Figure S2
A
Zfp281-/- ESCs:
- EV Rescue
ZFP281
Actin
B
Oct4
Nanog
Sox2
Gata4
Gata6
Sox17
mRNA Exp.
P0 P1 P3
C
ESC-signature genes
XEN-signature genes
FPKM
P=n.s. P=n.s. P=1.41e-8
P=n.s. P=0.045 P=2.2e-16
ESC (P0) P1 P3 XEN
Zfp281-/-
Zfp281-rescue
D
WT (Day 30)
Zfp281-/- (Day 30)
E
mRNA Exp.
Oct4 Nanog
Gata4 Gata6
WT (Day 0)
Zfp281-/- (Day 0)
WT (Day 30)
Zfp281-/- (Day 30)
XEN

Figure S3. ZFP281 is required for ESC-to-DE differentiation. (A) Schematic plot of WT and *Zfp281*$^{-/-}$ ESCs in differentiation to definitive endoderm cells (DEs) *in vitro*. (B) Expression of DE marker *Foxa2* is highly activated in this DE differentiation protocol. (C-D) Phase contrast microscope images (C) and cell viability assay (D) of WT and *Zfp281*$^{-/-}$ ESCs in ESC-to-DE differentiation. Data were collected at Day2, Day4, and Day5. The PI/AnnexinV double negative population (Q4) indicates the live cells, and quantification is shown in the right panel of (D).

Figure S3

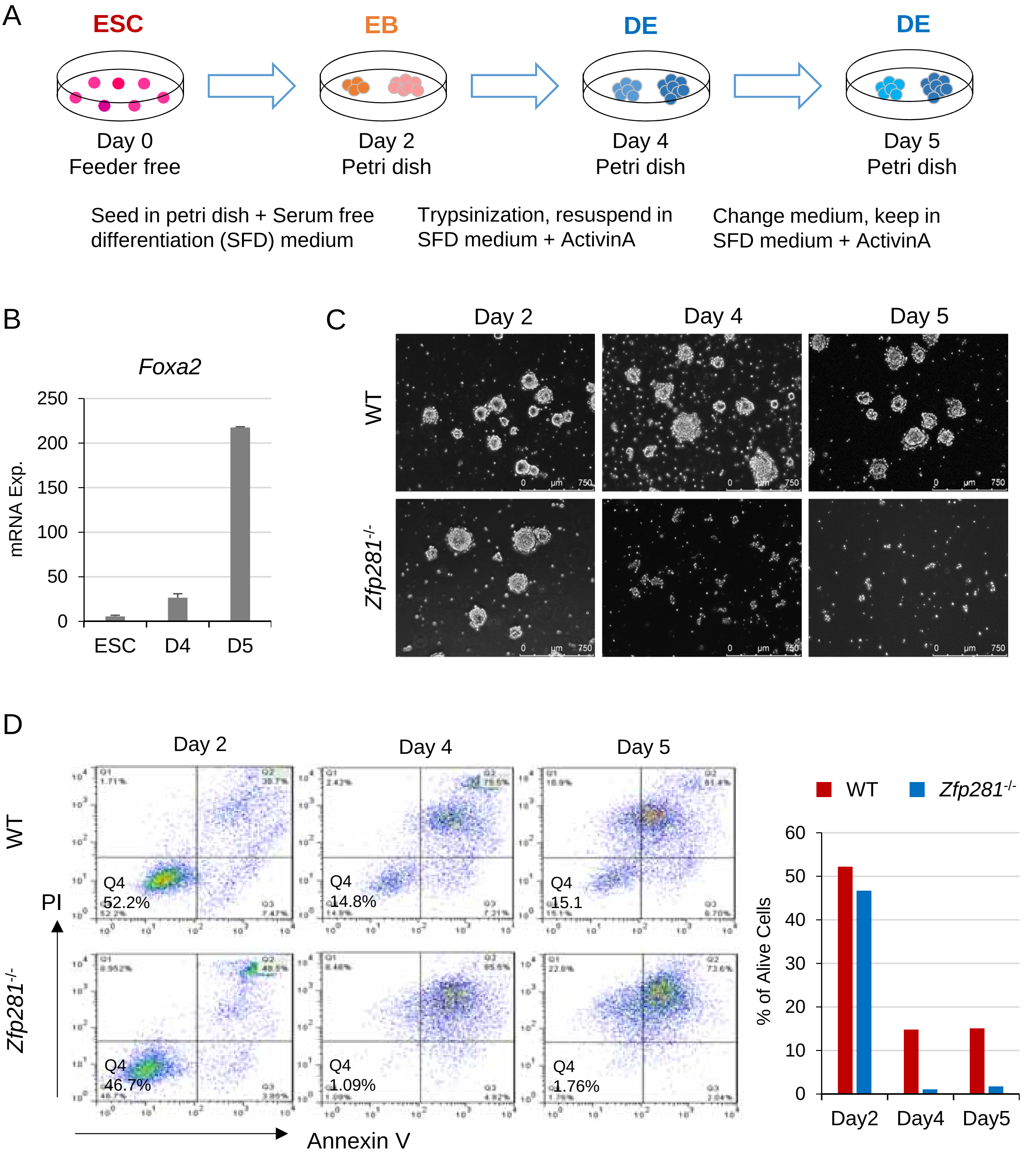
A
ESC
EB
DE
DE
Day 0
Feeder free
Day 2
Petri dish
Day 4
Petri dish
Day 5
Petri dish
Seed in petri dish + Serum free differentiation (SFD) medium
Trypsinization, resuspend in SFD medium + ActivinA
Change medium, keep in SFD medium + ActivinA
B
Foxa2
mRNA Exp.
250
200
150
100
50
0
ESC
D4
D5
C
Day 2
Day 4
Day 5
WT
Zfp281-/-
D
Day 2
Day 4
Day 5
WT
Zfp281-/-
PI
Annexin V
Q4
52.2%
Q4
14.8%
Q4
15.1
Q4
46.7%
Q4
1.09%
Q4
1.76%
WT
Zfp281-/-
% of Alive Cells
60
50
40
30
20
10
0
Day2
Day4
Day5

Figure S4. ZFP281 represses the PrE master genes through PRC2, but not TET1. (A-B) Box plots (A) for the expression of ESC-signature and XEN-signature genes, and expression (B) of *Gata4, Gata6, Sox17* in WT and *Eed*KO ESCs from RNA-seq data (Cruz-Molina et al., 2017). P-value is from a Mann-Whitney test. (C-D) Box plots (C) for the expression of ESC-signature and XEN-signature genes, and expression (D) of *Gata4, Gata6, Sox17* in WT and *Tet1*KO ESCs from RNA-seq data (Hon et al., 2014). P-value is from a Mann-Whitney test. (E) MeDIP-seq for the DNA methylation levels around promoters of *Gata4, Gata6, Sox17* in embryonic (ESC, EpiSC) and extraembryonic (XEN, TSC) tissues. Positions of the CpG Islands are labeled. Data are from (Senner et al., 2012).

Figure S4

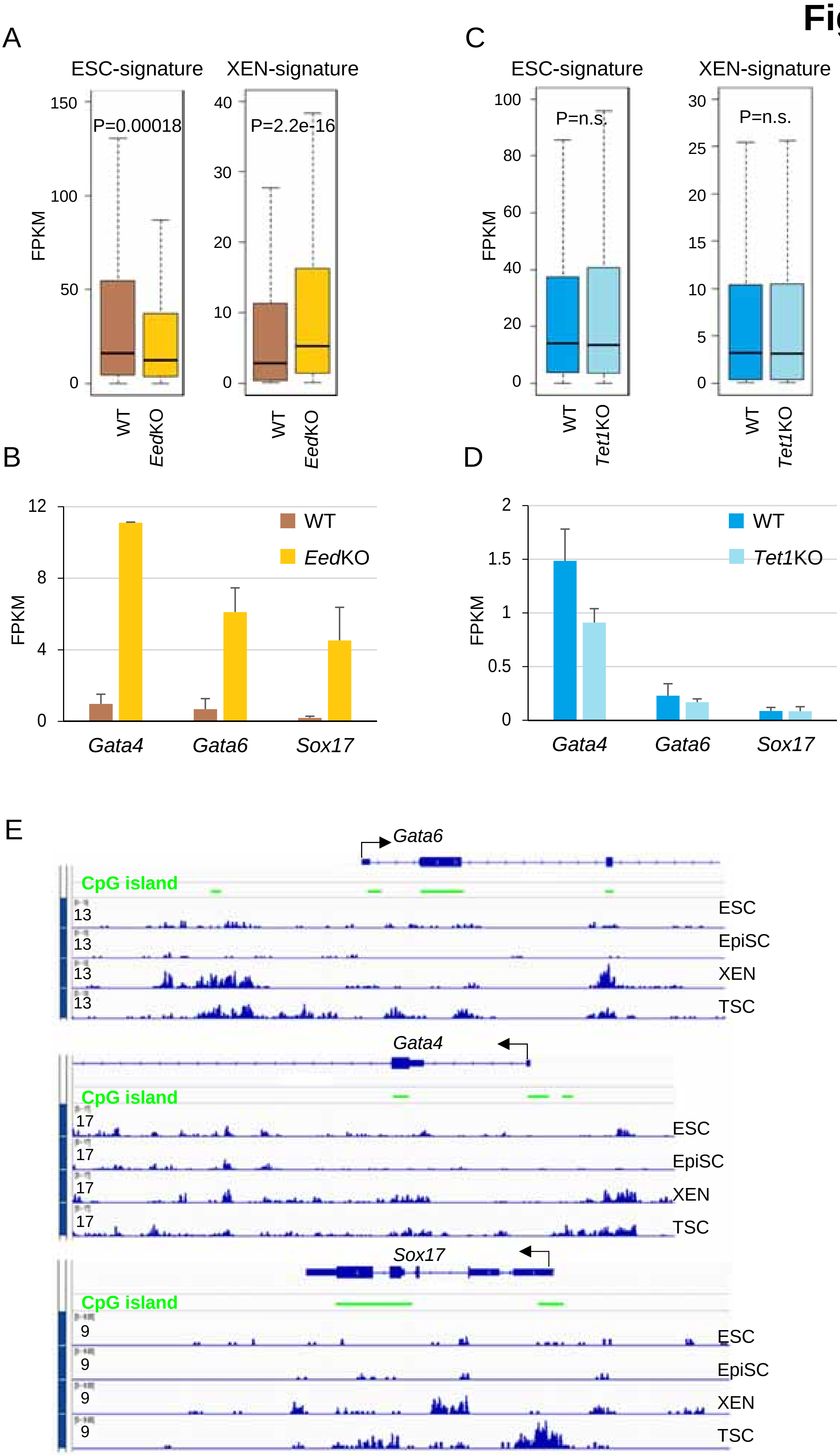

A
ESC-signature
XEN-signature
P=0.00018
P=2.2e-16
FPKM
150
100
50
0
40
30
20
10
0
WT
EedKO
WT
EedKO
C
ESC-signature
XEN-signature
P=n.s.
P=n.s.
FPKM
100
80
60
40
20
0
30
25
20
15
10
5
0
WT
Tet1KO
WT
Tet1KO
B
FPKM
12
8
4
0
WT
EedKO
Gata4
Gata6
Sox17
D
FPKM
2
1.5
1
0.5
0
WT
Tet1KO
Gata4
Gata6
Sox17
E
Gata6
CpG island
13
ESC
13
EpiSC
13
XEN
13
TSC
Gata4
CpG island
17
ESC
17
EpiSC
17
XEN
17
TSC
Sox17
CpG island
9
ESC
9
EpiSC
9
XEN
9
TSC

Figure S5. Additional ZFP281 and SUZ12 ChIP-seq analysis. (A) Overlap of the ZFP281 ChIP-seq peaks in ESCs and XENs. (B) Mean intensity plots (RPM) and heatmaps of SUZ12 and H3K27me3 ChIP-seq in ESCs and XENs enriched at all TSS sites. H3K27me3 ChIP-seq in ESCs is from (Cruz-Molina et al., 2017).

Figure S5

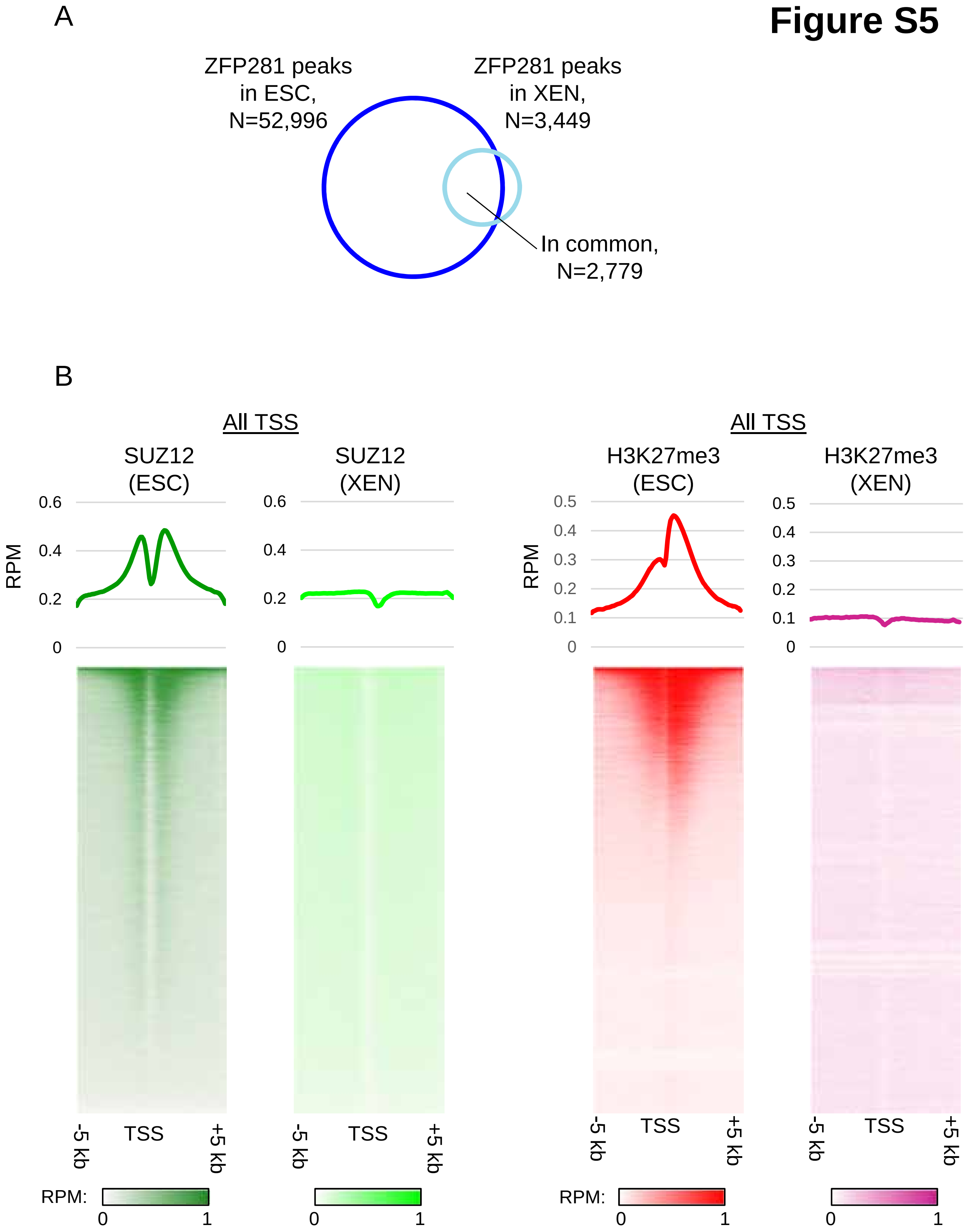

A
ZFP281 peaks in ESC, N=52,996
ZFP281 peaks in XEN, N=3,449
In common, N=2,779
B
All TSS
SUZ12 (ESC)
SUZ12 (XEN)
All TSS
H3K27me3 (ESC)
H3K27me3 (XEN)
RPM
0.6
0.4
0.2
0
0.5
0.4
0.3
0.2
0.1
-5 kb
TSS
+5 kb
RPM:
0
1

Figure S6. ZFP281 is dispensable for XEN maintenance. (A) Phase contrast microscope images of empty vector (EV) and *Zfp281* shRNA-treated XENs cells (on feeders). (B) Western blot analysis of ZFP281, GATA6, and SOX17 in XENs with EV and two *Zfp281* shRNA treatments. (C) Immunostaining of ZFP281 together with either GATA6 (left) or SOX17 (right) in XEN cells. (D) qRT-PCR analysis of *Zfp281*, PrE (*Gata4, Gata6, Sox17*) and pluripotency (*Oct4, Nanog*) transcripts in XENs upon *Zfp281* knockdown.

Figure S6

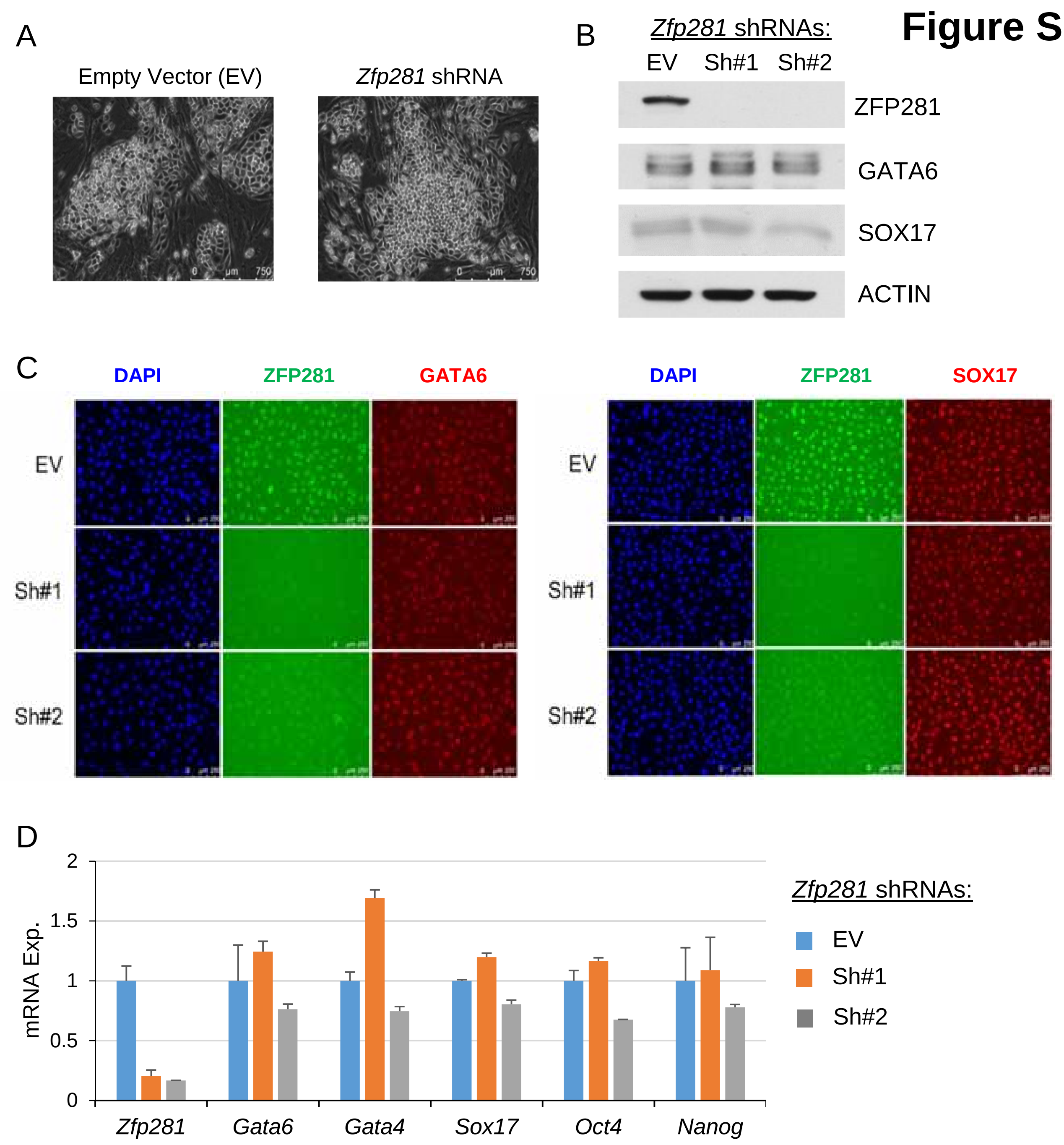
A
Empty Vector (EV)
Zfp281 shRNA
0 μm 750
B
Zfp281 shRNAs:
EV Sh#1 Sh#2
ZFP281
GATA6
SOX17
ACTIN
C
DAPI
ZFP281
GATA6
SOX17
EV
Sh#1
Sh#2
D
mRNA Exp.
2
1.5
1
0.5
0
Zfp281
Gata6
Gata4
Sox17
Oct4
Nanog
Zfp281 shRNAs:
EV
Sh#1
Sh#2
